# ADHD and schizophrenia: Mere prodromal variant or homogeneous subgroup?

**DOI:** 10.1016/j.scog.2025.100374

**Published:** 2025-06-18

**Authors:** J.B. Schulze, F. Simnacher, T.J. Müller, J. Kirchebner, F. Quatela, C. Mikutta, S. Euler, R. von Känel, M.P. Günther

**Affiliations:** a Department of Consultation-Liaison Psychiatry and Psychosomatic Medicine, University Hospital Zurich, University of Zürich, Switzerland; b Privatklinik Meiringen, Willigen, 3600 Meiringen, Switzerland; c Department of Forensic Psychiatry, University Hospital of Psychiatry Zurich, Zurich, Switzerland

**Keywords:** Schizophrenia spectrum disorder, Attention deficit hyperactivity disorder, Psychiatric comorbidities, Psychiatric disorders, Explorative analysis, Latent class analysis

## Abstract

**Introduction:**

Attention deficit hyperactivity disorder (ADHD) diagnosed in childhood is associated with a relative risk of 4.74 (95 % CI, 4.11–5.46) for developing schizophrenia spectrum disorder (SSD) later in life; if other comorbidities exist the risk is 2.1-fold higher. There is no guideline on treating ADHD in SSD and no research on the effect of this combination on length of inpatient treatment, type of pharmacotherapy and employment status. This study aims to further explore the role of ADHD in SSD.

**Methods:**

Latent Class Analysis (LCA) uses no a priori assumptions in testing for homogeneous subgroups within a data sample of 2871 inpatient treatment cases of SSD from three psychiatric hospitals. Data was extracted from case files and statistical reports to the federal statistical office.

**Results:**

Two subgroups are identified. One primarily consists of individuals with SSD and ADHD (estimated population size of 3 %). In comparison to the other subgroup with SSD and no ADHD (97 %), these individuals more frequently have other mental comorbidities, especially substance use disorders, are unemployed and about half are administered stimulants. All studied individuals were administered antipsychotics and length of inpatient stay was similar in both subgroups.

**Conclusion:**

ADHD and SSD define a subgroup of individuals with specific treatment needs and additional burden of disease. ADHD is more than an initial misdiagnosis or random precursor disease of SSD. Treating psychiatrists seem to frequently administer stimulants.

## 1 Introduction

Schizophrenia spectrum disorders (SSD) and attention-deficit/hyperactivity disorder (ADHD) have both been recognized as neurodevelopmental disorders (Kalin, 2024) affecting attention and memory functions (Armita et al., 2025; Egeland, 2007; Oie et al., 1999). In both disorders, poor cognitive performance is retrospectively often the first symptom (Oie et al., 1999) – even if ignored initially. Unsurprisingly, there have been cases in which early cognitive symptoms of a primary psychotic disorder were mistakenly attributed to the more vogue ADHD with stimulant prescription exacerbating (or even causing) positive symptoms (Karatekin et al., 2010; Shyu et al., 2015; Studerus et al., 2018). Yet, recent research suggested a more complex relationship between ADHD and development of SSD than the mere “misdiagnosis hypothesis”. A systematic review concluded 23 % of individuals with SSD experience some symptoms of ADHD (Arican et al., 2019). A systematic review and meta-analysis (1.85 million participants) indicated ADHD diagnosed in childhood is associated with a relative risk of 4.74 (95 % CI, 4.11–5.46) of developing schizophrenia later in life (Nourredine et al., 2021). Individuals with ADHD and another psychiatric comorbidity had a 2.1-fold higher risk to be diagnosed with schizophrenia in comparison to those without psychiatric comorbidities (Jeon et al., 2023). Thus, authors concluded proper treatment of comorbid conditions may prevent progression to SSD. Yet, comorbidities are so common in SSD, some argue them to be an integral part of the diagnosis (Bermanzohn et al., 2000), or that SSD should even “trump” other diagnoses, such as depression or anxiety (Buckley et al., 2009) – or ADHD? Thus, the question remains if ADHD is but one possible prodromal symptom of SSD with little further relevance once SSD is diagnosed or defines a subgroup of individuals with specific treatment needs.

Proposed explanations for the association between ADHD and SSD include common genetic root causes (e.g. chromosomal deletions and duplications), social environmental agents (e.g. obstetric complications, preterm birth, low birth weight), other environmental influences, prenatal developmental factors (e.g. overlapping brain circuits in the mesolimbic and mesocortical systems), and even psychostimulant treatment (Nourredine et al., 2021). For the latter, it remains unclear whether certain individuals overused stimulants, have lower thresholds for psychosis triggered by stimulants, or would have developed psychosis regardless of stimulants (Toba-Oluboka and Dempster, 2024). While overuse of prescribed stimulants is reported to be low (0.6 %) (Blanco and Surman, 2024), the psychotic potential of therapeutic doses may be augmented by concomitant substance use. Substance use disorders (SUDs) are common in both ADHD and SSD with 21 % (Rohner et al., 2023) and up to 50 % (Hunt et al., 2018), respectively, but have not been explored as a mediator.

Beyond diagnostic and aetiologic questions, there is no consensus on the treatment of co-occurring SSD and ADHD. For certain patients with SSD, the use of stimulants may potentially improve (hard to treat) negative symptoms without worsening positive symptoms (Lindenmayer et al., 2013). Yet, in others stimulants at therapeutic doses trigger psychotic symptoms independent of patients' current psychopathological state and despite concomitant administration of antipsychotics (Lieberman et al., 1987). There are no specific guidelines regarding the management of ADHD in individuals with SSD, but most guidelines recommend treatment of comorbidities before treatment of ADHD (Toba-Oluboka and Dempster, 2024). From prior research, it remains unclear whether individuals with SSD and ADHD are different from those with SSD (and no ADHD) in terms of treatment needs, comorbidities and overall level of functioning.

The following retrospective exploratory study aims to examine if individuals with SSD and ADHD are a homogeneous subgroup within all individuals with SSD, rather than ADHD being a variant of the prodromal phase before manifest SSD. Latent Class Analysis (LCA) is used as an advanced machine learning approach to identify homogeneous subgroups within a data sample of 2871 inpatient treatment cases from three psychiatric hospitals. LCA employs no prior preconceptions on the number of homogeneous subgroups present in a data set. Thus, it seemed ideal in exploring the hypothesis that individuals with ADHD and SSD experience more mental comorbidities, especially SUD, have additional treatment needs, potentially a longer treatment duration and more difficulty to pursue employment.

## 2 Material and methods

### 2.1 Sources of data and preliminary processing

**Fig. 1:**
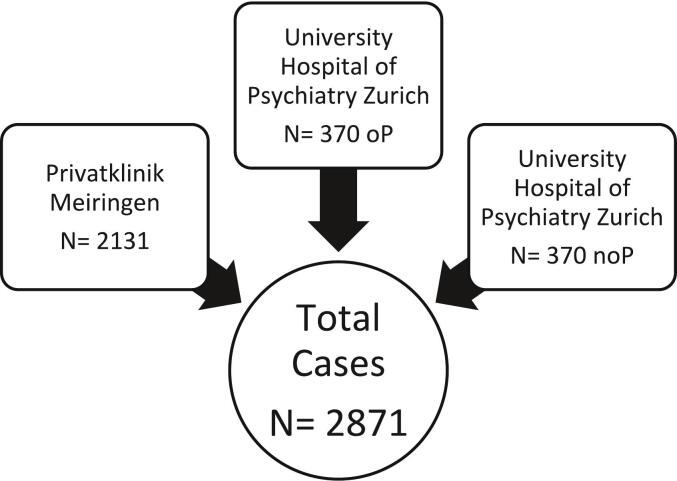
Study sample. Note. oP, offender patients; noP, non-offender patient.

Study design, data collection, its analysis and presentation follow the STROBE guidelines (von Elm et al., 2008). The following retrospective study is a multicenter study based on two primary data sources (see Fig. 1). The first data source included 2131 cases of inpatients diagnosed with SSD (F20.0-F25.9 according to ICD-10) treated at Privatklinik Meiringen, a large psychiatric hospital in central Switzerland, between 01.01.2008 and 31.12.2022. The data was derived from reports submitted to the department of statistics of the Swiss government and patient case files. Ethical approval was granted by the Cantonal Ethics Committee of Bern, Switzerland (BASEC No. 2023–02246).

The second data source consisted of two patient subgroups from the University Hospital of Psychiatry Zurich, one of the biggest psychiatric hospitals in northern Switzerland. The first subgroup included 370 offender patients diagnosed with SSD (F20.0-F25.9 according to ICD-10; 295.0–295.9 according to ICD-9) who had court-mandated inpatient treatment at the Centre for Inpatient Forensic Therapies. The second subgroup comprised 370 cases of non-offender patients with the same diagnostic inclusion criteria, who had been in inpatient treatment at the Centre for Integrative Psychiatry. The data of these two subgroups received ethics approval from the Cantonal Ethics Committee of Zurich, Switzerland (BASEC No. 2014–0480 and PB_2016–01903). The data stemmed from case files for treatments between 1982 and 2016, with most treatments (over 75 %) occurring after 2000.

The final dataset included a total of 2871 cases. The following variables were explored in the LCA: length of stay (length of inpatient treatment: long/short), ADHD diagnosed prior to SSD (yes/no), substance use disorder diagnosed (yes/no), other psychiatric comorbidity diagnosed (yes/no), antipsychotics (neuroleptics; ATC-codes N05A except N05AN) administered during inpatient treatment (yes/no), ADHD medication (stimulants; ATC-code N06BA) administered during inpatient treatment (yes/no) and employment status prior to admission to inpatient treatment according to the person treated (unemployed yes/no). An ADHD diagnosis was assumed to be present, if it was mentioned in prior medical reports (submitted when referring a patient or acquired during treatment). To create a binary variable for length of stay, the median value was calculated and used to classify cases into two categories: long (> median) and short duration (< median). Additionally, demographic data such as age and gender, as well as legal aspects, current medications and comorbidities were collected.

In the dataset, 8.43 % of patients had missing values concerning the diagnosis of ADHD. These cases were imputed using Multivariate Imputation by Chained Equations (MICE) under the Missing at Random (MAR) assumption, which is considered one of the best approaches, as it maintains variability and integrates uncertainty (Ambler et al., 2007). The process ran for 10 iterations, using predictors with correlations of *r* = 0.15–0.43, and binary variables were rounded to 0 or 1. Implementation used the IterativeImputer function from Python's scikit-learn library. With all other variables explored there was no missing data.

### 2.2 Statistical analysis

**Fig. 2:**
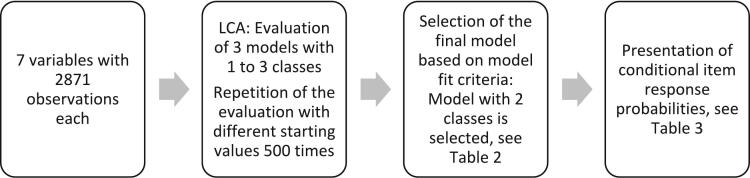
Flowchart of methodology. Note. LCA = latent class analysis.

The statistical analysis was conducted in several steps (see Fig. 2).

LCA was performed using the poLCA package implemented in R Studio, version 1.1.383. LCA is a (finite mixture) statistical method for identifying hidden subgroups with homogeneous characteristics (termed classes) within a sample of multivariate categorical data by analyzing patterns in the observed variables (Hagenaars and McCutcheon, 2002; Sinha et al., 2021). Specifically, the sample of 2871 cases is divided into distinct classes with homogenous characteristics within each class (Nylund-Gibson et al., 2007).

To identify the most parsimonious model, solutions with one to three classes were evaluated. To minimize the risk of local extrema and ensure model accuracy, each LCA model calculation was repeated 500 times with varying starting values. Maximum log-likelihood, Akaike information criterion (AIC), Bayesian information criterion (BIC) and entropy were considered when deciding for the most parsimonious final model, as has been suggested in prior literature (Günther et al., 2021a, Günther et al., 2021b; Nylund-Gibson et al., 2007; Schreiber, 2017). Log-likelihood is a measure of model fit, with higher values indicating a better alignment between the model and the observed data. AIC and BIC, on the other hand, evaluate how well a model fits the data by balancing its complexity against the sample size to avoid overfitting, with lower values indicating a better fit. Both criteria penalize models for additional parameters, but the BIC applies a stricter, sample size adjusted penalty to prevent overfitting and favours simpler models (Sinha et al., 2021). Therefore, BIC is often preferred, as it is considered a more reliable indicator of model fit (Nylund-Gibson et al., 2007), given that AIC has been shown to overestimate the number of classes in a model (Dziak et al., 2020). Entropy measures the separation between latent classes, with values ranging from 0 to 1; values close to 1 indicate clear differentiation between classes (Asparouhov and Bengt, 2019). However, for final model selection the absolute value should not be used, as overfitted models may also result in high entropy (Sinha et al., 2021).

## 3 Results

A detailed overview of the patient characteristics, including demographic and clinical variables such as age, gender, and psychiatric diagnoses, is provided in Table 1.

**Table 1:** Patient characteristics.

Socio-demographic item	All patients	
Age	Mean (years)	
	41,86	

Sex	N	Percent
Female	1128	39.3
Male	1743	60.7

Cumulative length of stay	N	Mean (days)
Low (≤ 36 Days)	1413	14.4
High (> 36 Days)	1458	314.5

Compulsory admittance	N	Percent
Yes	917	31.9
No	1954	68.1

Employment status	N	Percent
Employed	1463	50.9
Unemployed	1408	49.1

Pharmacotherapy	N	Percent
Stimulants (N06BA)^a^	60	2.1
Antidepressants (N06A)^a^	912	31.8
Antipsychotics (N05A)^a^	2540	88.5
Sedatives (N05C)^a^	1223	42.6

Schizophrenia spectrum disorder	N	Percent
Paranoid Schizophrenia (F20.0)	1506	52.5
Hebephrenic Schizophrenia (F20.1)	84	2.9
Schizoaffective Disorder (F25)	557	19.4
Other (F20.0-F25.9 except F20.0, F20.1 and F25)	724	25.2

ADHD (F90.0)	N	Percent
	101	3.5

Other mental disorders	N	Percent
Substance use disorder (F10-F19)	607	21.1
Cannabis use disorder (F12)	526	18.3
Opioid use disorder (F11)	194	6.8
Cocaine use disorder (F14)	219	7.6
Stimulant use disorder (F15)	127	4.4
Personality disorder (F60, F61, F68, F69)	191	6.6
Other psychiatric comorbidities (any other F-diagnosis)	590	20.5

Note.

a ATC-codes for substance.

The evaluation criteria outlined above identified the 2-class model as the most parsimonious solution. It achieved a lower BIC (17,238.79) in comparison to the 3-class model (17,257.13). This indicates that the 2-class model better balances model complexity and parsimony. Additionally, the 2-class model demonstrated adequate entropy (0.82), ensuring sufficient separation between subgroups, while having a lower risk of overfitting than the 3-class model with an entropy of 0.98 (see Table 2). From a clinical perspective the 2-class solution also seemed more comprehensive, as it divides the sample into a subgroup with ADHD and one without.

**Table 2:** Latent class model solutions and fit indices for 2-class and 3-class model.

Model solution	Number of estimated parameters	Residual degrees of freedom	Maximum LL	AIC	BIC	Entropy
2-class	15	112	−8559.677	17,149.35	**17,238.79**	0.815
3-class	23	104	−8536.997	17,119.99	17,257.13	0.977

Note. BIC highlighted with bold type indicating best model fit.

Abbreviations: LL, maximum log-likelihood; AIC, Akaike information criterion; BIC, Bayesian information criterion; entropy, measure of classification uncertainty.

**Fig. 3:**
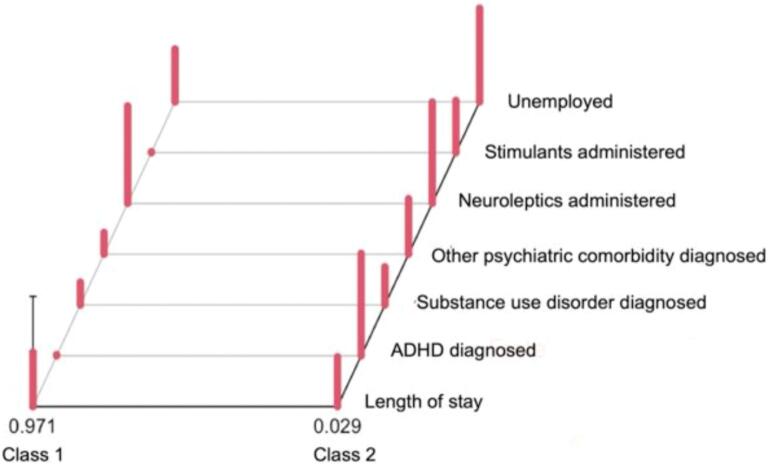
Graphical representation of conditional item response probabilities. Note. x-axis, class subgroups with estimated class population size; y-axis, probability of dichotomous characteristic to be present; z-axis, dichotomous variables explored.

Conditional item response probabilities were calculated for each class of the 2-class model (see Table 3 and Fig. 3). These probabilities represent the probability of each variable being positive within each class and offer insights into the characteristics that define each latent class. For example, the probability of a “low” length of stay in class 1 is 50.66 %, compared to 54.85 % in class 2. Interclass differences in item response probability (see Table 3) above 10 % have been suggested as clinically relevant in previous psychiatric research (Günther et al., 2021a, Günther et al., 2021b; Lau et al., 2021).

**Table 3:** Conditional item response probabilities of the two classes of the 2-class model.

Item	Class 1	Class 2	Interclass differences in item response probability
Estimated class population size	97.1 %	2.9 %	

Length of stay			
low	0.5066	0.5485	**0.0419**
high	0.4934	0.4515	

ADHD diagnosed			
No	**0.9901**	0.0709	0.9192
Yes	0.0099	**0.9291**	

Substance use disorder diagnosed			
No	0.7926	0.6532	0.1394
Yes	0.2074	0.3468	

Other psychiatric comorbidity diagnosed			
No	**0.8034**	0.4972	0.3062
Yes	0.1966	0.5028	

Neuroleptics administered			
No	0.1163	0.0808	**0.0355**
Yes	**0.8837**	**0.9192**	

Stimulants administered			
No	**0.9926**	0.5266	0.466
Yes	0.0074	0.4734	

Unemployed			
No	0.5203	0.1512	0.3691
Yes	0.4797	**0.8488**	

Note. Conditional item response probabilities above 0.80 are in bold type.

Interclass differences in item response probability below 10 % are in bold type.

In class 1, with an estimated class population share of 0.971, ADHD diagnoses are rare. Individuals are more likely to have moderate rates of SUDs and other psychiatric comorbidities. Most patients are administered antipsychotics, while the use of ADHD medication is uncommon. Employment is more prevalent in this group, and the length of stay is almost evenly distributed between short and long duration.

In class 2, with an estimated class population share of 0.029, most individuals are likely to have an ADHD diagnosis. A substantial proportion have a SUD and approximately half of the individuals are likely to have other psychiatric comorbidities. Administration of antipsychotics is widespread, and a significant proportion are also prescribed stimulants. In this class patients likely have a higher unemployment rate.

## 4 Discussion and conclusion

### 4.1 Discussion

Results confirm the hypothesis that ADHD in individuals with SSD is more than just another comorbidity but defines a subgroup of patients with an increased burden of disease. The final 2-class model derived via LCA suggests patients with SSD and ADHD (class 2; 3 % of patients with SSD) represent a distinct subgroup with specific clinical characteristics that differ from those with SSD (class 1; 97 %). Individuals with SSD and ADHD (class 2) show a significantly higher risk for comorbid mental disorders (50 % vs. 20 %), particularly SUDs (35 % vs. 21 %), are more likely to be unemployed (85 % vs. 48 %) and receive stimulant therapy (47 % vs. 1 %). Yet they have about the same probability to be administered antipsychotics (92 % vs. 88 %)^2^ and be hospitalized for longer than the median of 36 days (45 % vs. 49 %).

Results confirm prior research indicating patients with ADHD and SSD often have other mental comorbidities (Jeon et al., 2023). SUD was found to be one of the most common comorbidities in SSD, affecting up to 50 % of individuals (Hunt et al., 2018; Temmingh et al., 2021). Individuals with ADHD were also found to have an increased risk (odds ratio of 1.9) to develop a SUD in adulthood, with conduct disorder serving as a mediator (Brook et al., 2010; Wilens et al., 2011). Results of the present study indicate a higher risk of developing a SUD for patients with ADHD and SSD (35 %) in comparison to those with SSD (21 %), thus hinting that risk factors for SUD associated with SSD and ADHD alone may add up in those affected by SSD and ADHD. Again, this seems to strengthen the hypothesis that the combination of SSD and ADHD defines a specific subgroup of individuals rather than ADHD being one of many comorbidities often preceding SSD. The overall lower prevalence of SUDs (21.1 %) (see Table 1) may be due to convenience sampling and unreported SUDs (see limitations). Factors increasing the risk for SUDs in SSD are clinically relevant, as earlier studies reported that SUD in patients with SSD can adversely affect the course of the illness, leading to higher morbidity and mortality, potentially exacerbating positive symptoms, increasing hospitalization rates and reducing treatment adherence (Green et al., 2007; Hunt et al., 2018; Large et al., 2014; Schmidt et al., 2011). Also, it is well established, that SUDs in combination with SSD increases the risk of violent behavior significantly (OR of 8.9 versus 2.1) (Fazel et al., 2009).

The overall high unemployment rate (49 %,

Table 1) is consistent with earlier literature reporting an elevated risk of being unemployed for individuals with SSD (Christensen et al., 2022; Majuri et al., 2023). The even higher unemployment rate in patients with SSD and ADHD (85 %) may be due to the combination of cognitive deficits due to SSD and ADHD. As earlier research indicated, individuals with SSD were more impaired in visual memory than those with ADHD alone, while the latter were more impaired in working memory and maintaining attention than those with SSD (Oie et al., 1999). In another study, patients with ADHD had deteriorating attention over time, while patients with SSD (and no ADHD) experienced a training effect (Egeland, 2007). Direct assessment of cognitive performance in patients with SSD and ADHD would assist in better identifying individual challenges and in meeting individual treatment needs. Again, results indicate ADHD in combination with SSD increases the burden of disease in terms of a significantly higher unemployment rate – likely to be due to increased cognitive impairment. From a clinical perspective, this highlights higher needs for social support work and other interventions in patients with SSD and ADHD to enable them to integrate into the work force. In that context it would be interesting to explore the effect of subgroup specific medication (e.g. stimulants), psychotherapeutic and psychosocial interventions (including longer inpatient treatment) on cognitive functioning in patients with SSD and ADHD.

In the present study, increased needs in patients with ADHD and SSD, as outlined above, stand in contrast to the almost identical lengths of stay in this subgroup in comparison to individuals with SSD (45 % vs. 49 % were hospitalized for >36 days). This is also despite prior research suggesting more mental comorbidities in patients with SSD lead to prolonged hospitalizations (Kessler and Lev-Ran, 2019). However, a recent study revealed patients with SSD and SUD typically had shorter hospital stays, but were more frequently hospitalized (Burrer et al., 2024). The sample of patients with SSD and ADHD studied here (101 cases, Table 1) is too small and lacked relevant data to explore like explanation. However, of the 52 individuals with SSD and ADHD treated at one of the three psychiatric hospitals included, only 9 were rehospitalized at the same institution during the period of enquiry (2008–2022). Demographic factors, including ethnicity, region (Bessaha et al., 2017), gender, employment status (Goga and Marais, 2024) and clinical factors, such as illness severity, the presence of general medical comorbidities (Bessaha et al., 2017), treatment resistance (Goga and Marais, 2024), adherence to antipsychotic treatment (Barliana et al., 2023) and previous admission history (Velelekou et al., 2022) have been shown to significantly affect length of stay. Systemic factors, such as the type of admission (compulsory or voluntary) and the availability of social support networks, further influence the duration of inpatient care (Velelekou et al., 2022). Thus, overall further research is needed on whether longer hospitalizations of patients with SSD and ADHD would help them to obtain better employment rates/higher levels of cognitive functioning and social integration, or if other (e.g. pharmacological) interventions are needed.

Results of the present study evidenced patients in both subgroups are likely to be treated with antipsychotics, which is a standard treatment for SSD (Kaiser et al., 2018; Keepers et al., 2020) and thus evidence of a general adherence to treatment guidelines in the sample studied. In contrast, the probability of receiving ADHD medication is 47 % in those with SSD and ADHD and descriptive data reveal 60 % of them received stimulants during inpatient treatment. This may indicate that, despite the potential risks and controversy associated with stimulant use in individuals with psychotic disorders, as discussed above, stimulant therapy was perceived to have therapeutic value for patients with SSD and ADHD in many cases. On this, further research is needed.

In summary, individuals with both SSD and ADHD appear to represent a distinct group within the SSD population. Patients with both disorders appear to be more severely affected, evident in the higher probability for comorbid mental disorders (including SUDs) and unemployment. These findings underscore the need for further research on tailored and integrated treatment approaches to meet specific treatment needs of individuals with both disorders.

### 4.2 Limitations

Further limitations of this study, aside from those mentioned above, relate to its retrospective cross-sectional explorative nature. This kind of analysis limits our ability to draw definitive conclusions about causality and observe long-term effects of treatment approaches. It limits the choice of variables we can explore. However, our primary objective was to introduce as little bias as possible in exploring whether ADHD defines a specific subgroup of patients with comorbid SSD. This is also evident in using LCA, as it has no a priori assumptions in determining the number of subgroups to be identified (in contrast to other statistical approaches comparing predefined subgroups). Validation studies are needed (also to increase the size of the sample of patients with SSD and ADHD studied), before conclusions can inform clinical decisions. We used a multicenter design and included a forensic psychiatric sample and patients with involuntary inpatient admittance in an attempt to maximize generalizability. Yet, individuals with mild forms of SSD (and ADHD) not requiring inpatient treatment are missing from the sample. Similarly, severe forms of therapy resistant patients refusing inpatient treatment or being held in prisons may be underrepresented. The multicenter design also introduces variability in clinical practices across the different treatment centers, which may be of particular relevance in light of the controversy surrounding treatment with stimulants in patients with SSD. Data stemmed from different (overlapping) time frames, during which treatment and event documentation practices may have evolved, but (ICD-10) diagnostic and inclusion criteria remained the same throughout.

Last, no information was available on how ADHD was diagnosed (e.g., through neuropsychological testing or clinical assessment alone), except that in Switzerland health care insurance requires ADHD to be diagnosed by a psychiatrist experienced with ADHD. Payment for stimulant prescriptions is usually refused, if no detailed assessment is available, thus minimizing the risk for misdiagnosis.

### 4.3 Conclusion

To our best knowledge, this is the first study to provide evidence that ADHD in combination with SSD comprises a specific subgroup of individuals in comparison to individuals with SSD (even if in combination with other mental disorders). SSD does not seem to “trump” an earlier ADHD diagnosis. Individuals with SSD and ADHD have an increased burden of disease: They experience higher unemployment rates and more additional mental comorbidities, including SUDs. Current inpatient treatment tends to offer additional stimulant therapy in almost half of cases, but length of stay is similar to those with no additional ADHD. Further research may explore the effectiveness of subgroup specific pharmacological and psychosocial interventions (tertiary prevention). From a clinical perspective, it would be interesting to screen patients with SSD for ADHD, especially once better guidelines on subgroup specific treatment protocols have been developed.

## CRediT authorship contribution statement

**J.B. Schulze:** Writing – review & editing, Software, Formal analysis, Data curation. **F. Simnacher:** Writing – original draft, Project administration, Methodology, Investigation, Formal analysis, Data curation, Conceptualization. **T.J. Müller:** Writing – review & editing, Supervision. **J. Kirchebner:** Writing – review & editing, Data curation. **F. Quatela:** Writing – review & editing, Formal analysis, Data curation. **C. Mikutta:** Writing – review & editing, Project administration. **S. Euler:** Writing – review & editing, Supervision. **R. von Känel:** Writing – review & editing, Supervision. **M.P. Günther:** Writing – original draft, Validation, Resources, Project administration, Methodology, Investigation, Data curation, Conceptualization.

## Ethics statement

The authors affirm that all procedures involved in this research adhere to the ethical standards set by national and institutional committees responsible for human research and adhere to the Declaration of Helsinki. The study was approved by the Cantonal Ethics Committee of Bern, Switzerland (BASEC No. 2023–02246) and the Cantonal Ethics Committee of Zurich, Switzerland (BASEC No. 2014–0480 and PB_2016–01903).

## Funding statement

This research did not receive any specific grant from funding agencies in the public, commercial, or not-for-profit sectors.

## Declaration of competing interest

The authors state that they have no conflicting interests. No generative AI and AI-assisted technologies were used.

## Data Availability

The data supporting the findings of this study are available on reasonable request from the corresponding author. The data are not publicly available due to privacy or ethical restrictions.

## Footnotes

2 In Switzerland, patients with SSD have the right to refuse antipsychotic treatment and legal barriers to administer medication against their will are high (even in cases of compulsory inpatient admittance).
